# Discriminative Utility of Apelin-to-NT-Pro-Brain Natriuretic Peptide Ratio for Heart Failure with Preserved Ejection Fraction among Type 2 Diabetes Mellitus Patients

**DOI:** 10.3390/jcdd9010023

**Published:** 2022-01-12

**Authors:** Alexander A. Berezin, Ivan M. Fushtey, Alexander E. Berezin

**Affiliations:** 1 Internal Medicine Department, Medical Academy of Postgraduate Education, 69096 Zaporozhye, Ukraine; lunik.mender@gmail.com (A.A.B.); zmapo15@gmail.com (I.M.F.); 2 Internal Medicine Department, State Medical University, 69096 Zaporozhye, Ukraine

**Keywords:** heart failure with preserved election fraction, type 2 diabetes mellitus, apelin, prediction

## Abstract

Background: Apelin is a regulatory vasoactive peptide, which plays a pivotal role in adverse cardiac remodeling and heart failure (HF) with reduced ejection fraction. The purpose of the study was to investigate whether serum levels of apelin is associated with HF with preserved election fraction (HFpEF) in patients with T2DM. Methods: The study retrospectively involved 101 T2DM patients aged 41 to 62 years (48 patients with HFpEF and 28 non-HFpEF patients). The healthy control group consisted of 25 individuals with matched age and sex. Data collection included demographic and anthropometric information, hemodynamic performances and biomarkers of the disease. Transthoracic B-mode echocardiography, Doppler and TDI were performed at baseline. Serum levels of N-terminal pro-brain natriuretic peptide (NT-proBNP) and apelin were measured by ELISA in all patients at the study entry. Results: Unadjusted multivariate logistic model yielded the only apelin to NT-proBNP ratio (OR = 1.44; *p* = 0.001), BMI > 34 кг/м^2^ (OR = 1.07; *p* = 0.036), NT-proBNP > 458 pmol/mL (OR = 1.17; *p* = 0.042), LAVI > 34 mL/m^2^ (OR = 1.06; *p* = 0.042) and E/e’ > 11 (OR = 1.04; *p* = 0.044) remained to be strong predictors for HFpEF. After obesity adjustment, multivariate logistic regression showed that the apelin to NT-proBNP ratio < 0.82 × 10^−2^ units remained sole independent predictor for HFpEF (OR = 1.44; 95% CI: 1.18–2.77; *p* = 0.001) HFpEF in T2DM patients. In conclusion, we found that apelin to NT-proBNP ratio < 0.82 × 10^−2^ units better predicted HFpEF in T2DM patients than apelin and NT-proBNP alone. This finding could open new approach for CV risk stratification of T2DM at higher risk of HF.

## 1. Introduction

Heart failure (HF) with preserved ejection fraction (HFpEF) is a highly prevalent and intractable phenotype of HF which commonly occurs in patients with hypertension, metabolic diseases including type 2 diabetes mellitus (T2DM), abdominal obesity, metabolic syndrome and female gender [1,2]. Despite all-cause mortality risk and economic burden being found to be higher in HF with reduced (HFrEF) and mildly reduced (HFmrEF) ejection fraction than in HFpEF, cardiovascular (CV) death and HF-related complications, such as primary urgent hospital admission and early re-hospitalization, in patients with HFrEF/HFmrEF have exhibited a strict resemblance with those who had HFpEF [3,4].

The development of adverse cardiac remodeling and progression of HFpEF is suggested to be secondary to changes at the cellular structure and molecular metabolism of cardiac myocytes and cardiac cellular environment directly related to impaired glucose tolerance, insulin resistance, lipid toxicity, oxidative stress injury, adipocyte dysfunction and microvascular inflammation [5,6]. These particularities of HFpEF pathogenesis may explain serious limitations of conventional biomarker-shaped predictive scores based on measurement of natriuretic peptides (NPs) in patients at risk of HFpEF [7,8]. Indeed, NPs, such as N-terminal fragment of pro-brain natriuretic peptide (NT-proBNP) and BNP, along with high-sensitive cardiac troponins (hs-cTn) remain the dominant predictors of all-cause and CV mortality for patients with HFrEF/HFmrEF, but their importance in predicting events among HFpEF individuals especially having T2DM has not been extensively elucidated and continue to consider as non-optimal [9,10]. In this context, discovery of new biomarkers reflecting key stages of the pathogenesis of HFpEF with the aim of identifying patients with unfavorable functional profiles and higher risk of poor clinical outcomes appears promising [11].

Apelin is a regulatory vasoactive peptide, which is a ligand of the APJ receptor that belongs to the family of G protein-coupled receptors [12]. It exists in various active forms, such as apelin-36, apelin-17, apelin-13 and the pyroglutamated form of apelin-13 [13]. Apelin is extensively expressed in numerous organs and tissues and plays a pivotal role in adverse cardiac remodeling, while its molecular action seems to be controversial [14]. Being involved in the up-regulation of both myocardial and vasculature expressions of collagen-II and platelet-derived growth factor receptor β, apelin exerts pro-fibrotic potency and alleviates angiogenesis, but the apelin-APJ axis can contribute also anti-fibrotic, anti-proliferative, anti-ischemic and vasoactive effects through multiple pathways, such as suppression of the transforming growth factor-β1 expression, inhibition of phosphatidylinositol 3-kinase/protein kinase B and activation of AMPK-KLF2-eNOS-NO signaling [15,16]. In addition, apelin reduced oxidative damage by suppression of mitochondrial ROS-triggered injury and mitochondria apoptosis, and prevented ischemia/reperfusion damage along with a potentiation of inflammatory responses resulting of the activation of NF-κB and NLRP3 inflammasome release [17,18].

Circulating levels of apelin were found to be significantly reduced in patients with HFrEF when compared with those who had HFpEF or had no HF whatsoever, although patients with either abdominal obesity or T2DM have demonstrated elevated levels of the peptide [19,20]. Therefore, the levels of apelin have been noticed to be up regulated following potentially reversible adverse cardiac remodeling [21]. There was a large body of evidence regarding the fact that apelin influenced direct inotrope, vasodilator and diuretic effects and exerted crucial cardiac and vascular protective effects against angiotensin-II- and aldosterone-induced injuries and thereby counteracted with activated renin-angiotensin-aldosterone system in HF and acute myocardial infarction [22,23,24]. Previous clinical study has yielded that apelin was able to improve the predictive ability of the MAGGIC (Meta-Analysis Global Group In Chronic Heart Failure) and HFSS (Heart Failure Survival Score) scales adding new prognostic information to NT-proBNP in patients with severe HFrEF [25]. However, there is not fully clear whether apelin is able to predict HFpEF among T2DM patients, while there was conflicting evidence regarding an inverse association between the levels of apelin and mortality rate in HFrEF [26,27]. The purpose of the study was to investigate whether serum levels of apelin predict HFpEF in patients with T2DM.

## 2. Materials and Methods

### 2.1. Study Design and Cohort Identifications

One hundred and one participants aged 41 to 62 years (25 healthy volunteers and 76 T2DM patients) were retrospectively recruited to the study from the database consisted of the outpatients and in-patients who were treated in the private hospital Vita-Centre (Zaporozhye, Ukraine) from October 2020 to October 2021. Among 76 T2DM individuals, 48 patients had established HFpEF and 28 subjects did not have HFpEF.

**Figure 1:**
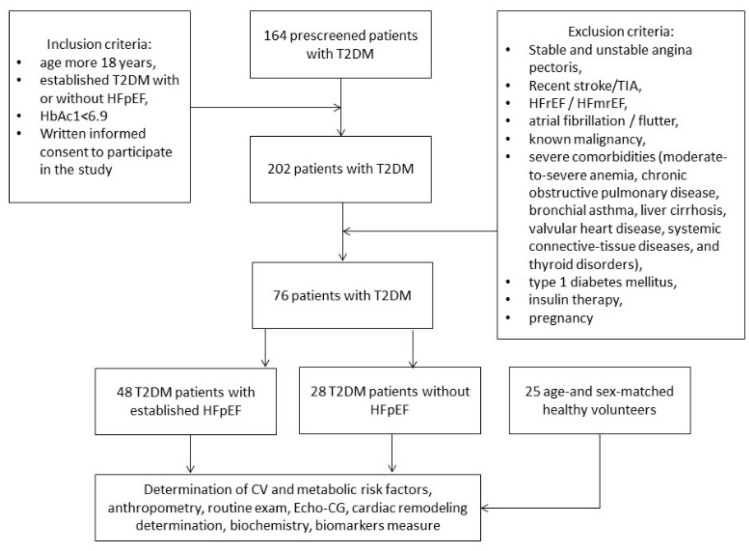
The flow chart of the study design.

Inclusion criteria were age more 18 years, established T2DM with or without HFpEF, well control for hyperglycemia (HbAc1 < 6.9%), consent to participate in the study. Exclusion criteria were the following: stable and unstable angina pectoris, recent stroke and transient ischemic attack, HFrEF/HRmrEF, atrial fibrillation, known malignancy, severe comorbidities (anemia, chronic obstructive lung disease and bronchial asthma, liver cirrhosis, known valvular heart disease, systemic connective-tissue diseases and thyroid disorders), type 1 diabetes mellitus, insulin therapy and pregnancy. All participants from the control group were healthy, and none of them had a history of CVD, TIA/stroke, valvular heart disease, congenital heart disease, cardiomyopathy and chronic HF as determined by history taking, questionnaires and clinical examination. Flow chart of the study design is reported Figure 1.

### 2.2. Determination of Risk Factors and Comorbidities

T2DM was determined according to new ADA statement (2017) [28]. Dyslipidemia was diagnosed if total cholesterol (TC) level was above 5.2 mmol/L, and/or low-density lipoprotein cholesterol (LDL) level was above 3.0 mmol/L, and/or level of triglycerides (TG) was above 1.7 mmol/L according to with ECS dyslipidemia guideline (2016) [29]. Hypertension was diagnosed if systolic blood pressure (SBP) was >140 mm Hg, and/or diastolic blood pressure (DBP) >90 mm Hg according to ESC guideline on diagnostics and treatment of arterial hypertension (2018) [30]. All enrolled T2DM patients who have ever been treated with antihypertensive drugs (one antihypertensive drug and more) were considered as those who had hypertension. HFpEF was diagnosed according to the 2016 ESC guideline [31], which was a valid in the period of patients’ selection.

### 2.3. General Anthropometric, Clinical and Physical Examinations

Conventional anthropometric measurements (height, weight, waist circumference, hip-to-waist ratio and body mass index (BMI)) were performed. All patients underwent general clinical and physical examination, office blood pressure and heart rate measure, as well as ambulatory blood pressure monitoring at the study entry.

### 2.4. Concomitant Medications

Hyperglycemia was controlled using a combination of metformin in individual daily doses and SGLT2 inhibitor (empagliflozin 10 mg daily or dapagluflosin 10 mg daily) giving orally. All patients with HFpEF received ACE inhibitor (ACEI) or angiotensin-II receptor antagonist (ARB), beta-blockers in individually adjusted optimal daily doses. Loop diuretic was prescribed when fluid retention was determined. In case of hypertension office BP < 140/90 mm Hg and average daily BP < 130/80 mm Hg was mandatory reached with adding to mentioned above drugs (ACEI or ARB and beta-blockers) diuretics or calcium channel antagonist mainly amlodipine. Rosuvastatin (20–40 mg daily) and acetylsalicylic acid (75 mg daily) were also prescribed as concomitant medications.

### 2.5. Echocardiography and Doppler

Structural and functional parameters of the heart were determined by echocardiography using the diagnostic system “GE Medical Systems” (Germany) by phase sensor with modulated frequency of 2.5–3.0 MHz in B-mode in accordance with current recommendations of the European Association of Cardiovascular Imaging (EACVI) and the American Society of Echocardiography (ASE) [32]. Left ventricular (LV) ejection fraction was calculated using Simpson method [32]. LV myocardial mass (LVMM) were measured and the LVMM index (LVMMI) was calculated as the ratio of LVMM to the surface area of the body. LVMMI > 115 g/m^2^ in male and > 95 g/m^2^ in female were criteria of LV hypertrophy [33]. Left atrial volume was directly measure and then left atrial volume index (LAVI) was estimated. E/e’ ratio was calculated at baseline.

### 2.6. Estimating Glomerular Filtration Rate

Glomerular filtration rate (GFR) was calculated using CKD-EPI formula [34].

### 2.7. Insulin Resistance Determination

Insulin resistance was evaluated by an estimation of the Homeostatic Assessment Model of Insulin Resistance (HOMA-IR) using appropriate equation [35]:

HOMA-IR = fasting insulin (mU/L) × fasting glucose (mmol/L)/22.5

### 2.8. Biomarkers Measurement

Blood samples were drawn in the morning following overnight fasting (at 7–8 a.m.) into barcoded silicone test tubes. Then samples were centrifuged upon permanent cooling at 6000 rpm for 3 min and then plasma was collected to be immediately refrigerated. Each aliquot was stored at a temperature −70 °C.

In order to measure the levels of glycosylated hemoglobin (HbA1c) and fasting glucose in whole blood we used Roche P800 analyzer (Basel, Switzerland).

Fasting insulin levels were measured by chemiluminescence method using commercial kits manufactured by DRG (USA) using Roche P800 analyzer (Basel, Switzerland).

Fasting total cholesterol (TC), low LDL cholesterol, high density lipoprotein (HDL) cholesterol and triglycerides (TG) were measured direct enzymatic method (Roche P800 analyzer, Basel, Switzerland).

We performed commercial ELISA kits produced by Elabscience (Houston, TX, USA) to determine the levels of high sensitive C-reactive protein (hs-CRP), apelin, NT-proBNP and high-sensitive cardiac troponin T (hs-TrT) according to recommendation of the manufacturer. Labline-90 analyzer (Austria) and Elecsys 1010 analyzer (F. Hoffmann-La Roche Diagnostics, Mannheim, Germany) were used respectively for the measures. The inter and intra assay coefficients of variations for ELISA kits were ≤2.5% and <3%, respectively.

### 2.9. Statistical Analyses

Categorical variables were defined as counts and percentages and differences between the groups were assessed by the chi-squared test. Continuous variables were characterized by either mean ± standard deviation (SD) or median (Me) and inter quartile range (IQR), if data were normally or not normally distributed respectively. The chi-square test was applied for non-continuous variables. Kolmogorov–Smirnov test was used to test for normal distribution. Spearman correlation coefficient was used to ascertain the relationship between variables. ROC curve with the Youden’s J index—a measure of maximum potential effectiveness by integrating sensitivity and specificity—was used in the predictive model. Predictors for HFpEF were determined by univariate and multivariate logistic regression. Odds ratio (OR) and 95% confidence interval (CI) were reported for each predictor. Differences were considered significant at the level of statistical significance *p* < 0.05.

## 3. Results

### 3.1. Study Population and Baseline Characteristics

Patients from entire cohort were mainly man, had dyslipidemia, hypertension, smoking, abdominal obesity and LV hypertrophy (Table 1). Higher proportion of smokers, as well as patients having other conventional CV risk factors, such as dyslipidemia, abdominal obesity, microalbuminuria and LV hypertrophy, LV diastolic dysfunction were noticed in entire cohort compared to healthy volunteers. However, there were no significant differences between T2DM patients with HFpEF or non-HFpEF in the majority of demographic, clinical and hemodynamic performances apart from LVMMI, LAVI and E/e’, which were prominently higher in HFpEF patients compared to non-HFpEF individuals. Consequently, LV hypertrophy was occurred more frequently in HFpEF than in non-HFpEF patients.

Table 2 illustrates the fact that there were no substantial differences between T2DM patients with HFpEF or non-HFpEF in eGFR, HOMA-IR, fasting glucose, hs-CRP, levels of total cholesterol, triglycerides and high-density of lipoproteins, whereas levels of hs-cTnT were significantly higher, and levels of and LDL-C were markedly lowered in HFpEF patients compared with non-HFpEF patients.

**Figure 2:**
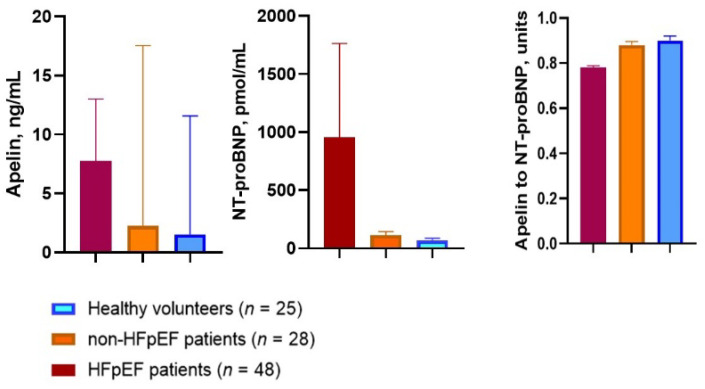
Apelin and NT-proBNP serum levels as well as the apelin-to-NT-proBNP ratio in T2DM patients with and without HFpEF in comparison with healthy volunteers. Abbreviations: HFpEF, heart failure with preserved ejection fraction; NT-proBNP, N-terminal fragment of brain natriuretic pro-peptide.

Serum levels of apelin and NT-proBNP were significantly increased in patients with HFpEF (7.74 ng/mL, 95% CI = 6.31–8.25 ng/mL and 954.8 pmol/mL, 95% CI = 476.2–87.4 144.9 1764.3 pmol/mL, respectively) when compared with non-HFpEF diabetics (2.26 ng/mL; 95% CI = 1.70–87.4 2.90 ng/mL; and 113.5 pmol/mL, 95% CI = 75.4–87.4 144.9 pmol/mL, respectively) and healthy volunteers (1.52 ng/mL 95% CI = 1.12–87.4 2.13 ng/mL and 67.8 pmol/mL, 95% CI = 49.1–87.4 pmol/mL; respectively) (Figure 2). On contrary, there were no substantial differences between diabetics with and without HFpEF in the levels of hs-CRP and hs-TrT.

### 3.2. Correlations between HOMA-IR and Anthropometric Parameters, Age, LV Hypertrophy and Biomarkers

It entire patient’ population HOMA-IR positively correlated with serum TG (r = 0.44; *p* = 0.001), WHR (r = 0.42; *p* = 0.001), waist circumference (r = 0.43; *p* = 0.001), BMI (r = 0.40; *p* = 0.002), LH hypertrophy (r = 0.40; *p* = 0.001), LAVI (r = 0.30; *p* = 0.001), serum levels of apelin (r = 0.32; *p* = 0.001) and age (r = 0.44; *p* = 0.012), and inversely with serum HDL-C (r = −0.34; *p* = 0.042), whereas there were no significant correlations to serum NT-proBNP (r = 0.11; *p* = 0.36) and hs-TrT (r = 0.09; *p* = 0.96).

In T2DM population without HFpEF HOMA-IR was significantly and positively correlated with WHR (r = 0.50; *p* = 0.001), waist circumference (r = 0.48; *p* = 0.001), BMI (r = 0.48; *p* = 0.002), LH hypertrophy (r = 0.42; *p* = 0.001), age (r = 0.44; *p* = 0.012), serum TG (r = 0.46; *p* = 0.001), hs-CRP (r = 0.38; *p* = 0.042) and NT-proBNP (r = 0.27; *p* = 0.036), apelin (r = 0.32; *p* = 0.022). Microalbumiuria (r = 0.30; *p* = 0.001), smoking (r = 0.24; *p* = 0.001). In addition, there were inverse correlations of HOMA-IR with serum HDL-C (r = −0.40; *p* = 0.02).

In T2DM patients with established HFpEF HOMA-IR demonstrated positive correlation with LH hypertrophy (r = 0.48; *p* = 0.001), BMI (r = 0.41; *p* = 0.001), LAVI (r = 0.36; *p* = 0.001), serum levels of TG (r = 0.37; *p* = 0.012), NT-proBNP (r = 0.46; *p* = 0.001), hs-TrT (r = 0.29; *p* = 0.032) and hs-CRP (r = 0.41; *p* = 0.014).

### 3.3. Correlations between Apelin and Other Variables

Apelin levels correlated positively with E/e’ (r = 0.38, *p* = 0.001), LV hypertrophy (r = 0.34, *p* = 0.001), BMI (r = 0.30, *p* = 0.001), HDL cholesterol (r = 0.28, *p* = 0.001), HOMA-IR (r = 0.32, *p* = 0.001) and age (r = 0.30, *p* = 0.001) and inversely with left ventricular ejection fraction (r = −0.34, *p* = 0.002). Serum levels of apelin did not correlate with the levels of both hs-TrT and NT-proBNP.

### 3.4. Other Correlations

We also found several positive correlations between the following variables, such as BMI and NT-proBNP (r = 0.33, *p* = 0.001), E/e’ and NT-proBNP (r = 0.32, *p* = 0.001), LAVI and NT-proBNP (r = 0.34; *p* = 0.001) and borderline inverse correlation between BMI and left ventricular ejection fraction (r = 0.22, *p* = 0.05) was noticed too.

### 3.5. The ROC Curve

**Figure 3:**
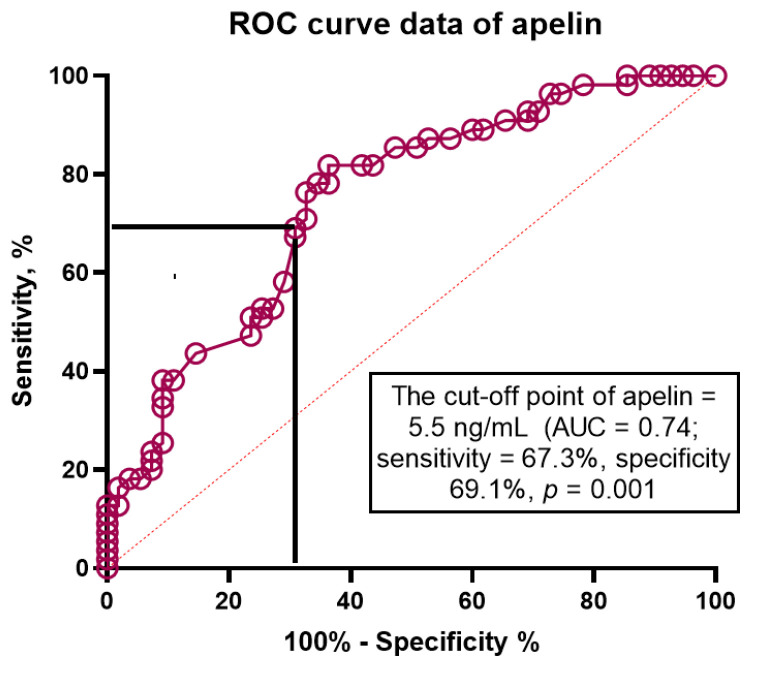

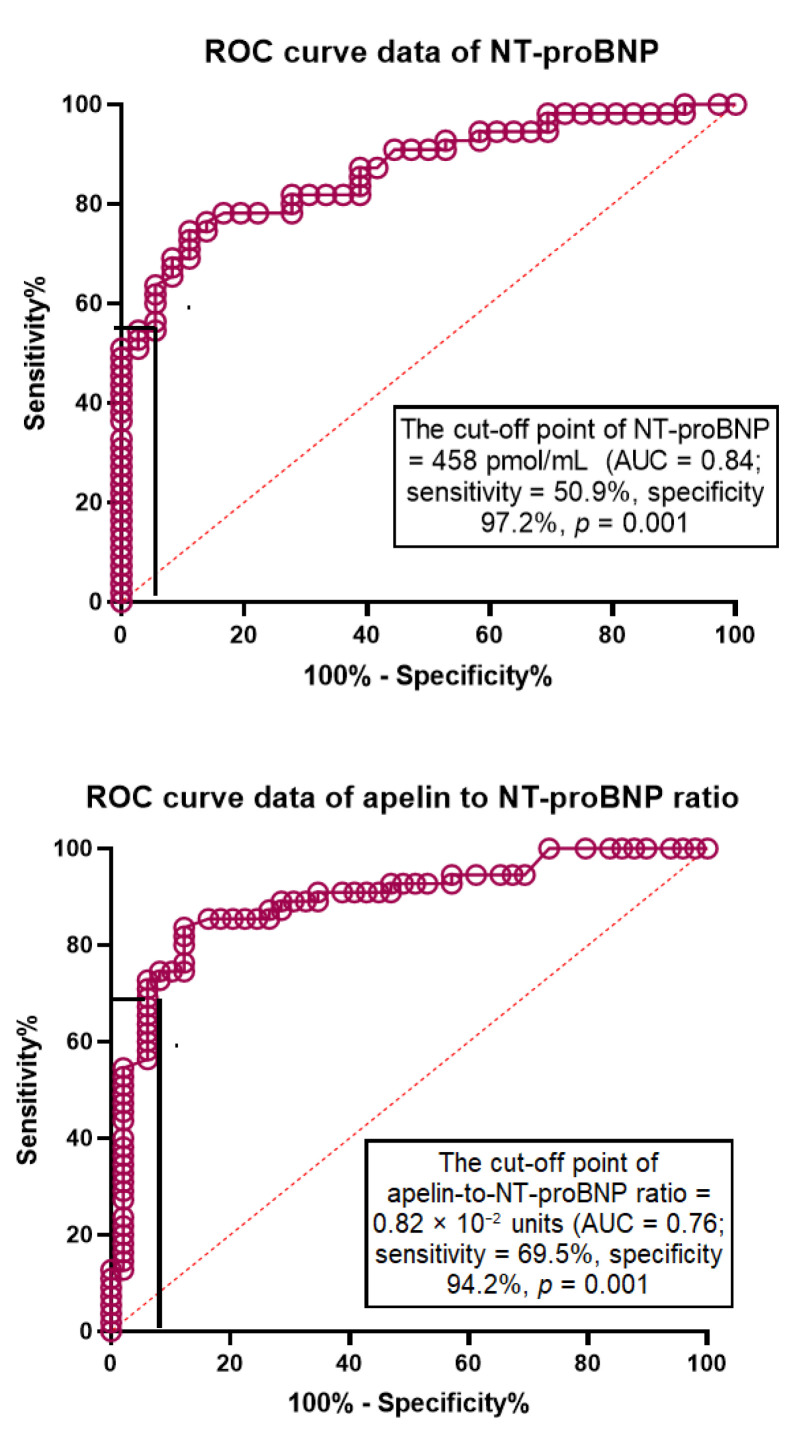
Apelin, NT-proBNP and Apelin to NT-proBNP ratio in prediction of HFpEF: Receiver operating characteristic curve analysis.

The ROC curve analysis (Figure 3) showed that the best fitted cut-off points for apelin and NT-proBNP were 5.5 ng/mL (area under curve [AUC] = 0.74, sensitivity = 67.3%, specificity = 69.1%; *p* = 0.001) and 458 pmol/mL (AUC = 0.84, sensitivity = 50.9%, specificity = 97.2%; *p* = 0.001), respectively. The well-balanced cutoff point for the apelin to NT-proBNP ratio to predict HFpEF was 0.82 × 10^−2^ units (area under curve = 0.76; sensitivity = 69.5% and specificity 94.2%, *p* = 0.001).

### 3.6. Univariate and Multivariate Logistic Regressions

Unadjusted univariate logistic regression showed that apelin to NT-proBNP ratio (OR = 1.32; *p* = 0.001), LV hypertrophy (OR = 1.16; *p* = 0.046), age (OR = 1.03; *p* = 0.048), NT-proBNP > 458 pmol/mL (OR = 1.24; *p* = 0.001), apelin > 4.5 ng/mL (OR = 1.06; *p* = 0.046), LAVI > 34 mL/m^2^ (OR = 1.20; *p* = 0.001) and E/e’ > 11 (OR = 1.12; *p* = 0.001) were found to be independent predictors for HFpEF in T2DM patients (Table 3). Pharmacological agents were not found to be predictors for the depending variable. Unadjusted multivariate logistic model yielded the only apelin to NT-proBNP ratio (OR = 1.44; *p* = 0.001), BMI > 34 кг/м^2^ (OR = 1.07; *p* = 0.036), NT-proBNP > 458 pmol/mL (OR = 1.17; *p* = 0.042), LAVI > 34 mL/m^2^ (OR = 1.06; *p* = 0.042) and E/e’>11 (OR = 1.04; *p* = 0.044) remained to be strong predictors for HFpEF.

After obesity adjustment multivariate logistic regression showed that the apelin to NT-proBNP ratio < 0.82 × 10^−2^ units remained the only independent predictor for HFpEF (OR = 1.44; 95% CI: 1.18–2.77; *p* = 0.001) HFpEF in T2DM patients.

## 4. Discussion

In this study, we first found that the apelin to NT-proBNP ratio < 0.82 × 10^−2^ units had independent from other biomarkers, such as LAVI, E/e’, LV hypertrophy and hs-TrT, discriminant potency for HFpEF in T2DM patients’ population. Although parameters that strongly describe the severity of LV diastolic dysfunction including LV hypertrophy, LAVI and E/e’, have previously demonstrated their clinical and prognostic relevance of determining the HF occurrence, HF admission and all-cause mortality in general population [36,37,38,39], HFpEF continues to be misdiagnosed in T2DM patients [40]. In this context, biomarkers’ models appear to be powerful multimodal diagnostic and predictive tools to identify T2DM patients with different degree of adverse cardiac remodeling and stratify them at the risk of HFpEF manifestation [41]. Having a myriad of underlying metabolic abnormalities, which have implicated in the development and progression of HFpEF related to T2DM, the combination of biomechanical stress biomarker (NT-proBNP) and regulatory peptide with inotropic ability (apelin) seems to be promising [42].

Herein we confirmed that serum levels of apelin and NT-proBNP were elevated in HFpEF patients compared non-HFpEF diabetics, but high variability of their concentrations substantially minimize predictive ability for HFpEF in T2DM population. Indeed, predictive ability of NT-proBNP for HFpEF remains to be challenged in T2DM patients due to increased proportion of patients having comorbidities (abdominal obesity and chronic kidney disease) that showed bidirectional influence on a clearance of natriuretic peptides [43]. In fact, impaired kidney function related to advanced T2DM-induced nephropathy was found to be strongly associated with increased circulating levels of NT-proBNP having a kidney clearance [44]. Yet, obesity upregulates an expression and circulating levels of neprilysin, which being an integral membrane-bound proteolytic metallopeptidase that disintegrates a wide spectrum of substrates including natriuretic peptides leads to increase circulating levels of NT-proBNP [45]. In addition, an accumulation of adipose tissue-increasing signaling pathway via the leptin receptor promotes an activation of both the sympathetic nervous system and renin–angiotensin–aldosterone system, which interplay with adverse cardiac remodeling and HF development [46]. Other pathogenetic mechanisms that underlies HF occurrence in T2DM patients are a stimulation of renal sympathetic nerves that interferes with adiponectin signaling, in turn cause over-activity of neprilysin, and thereby induce maladaptive impact of natriuretic peptides on tissues exacerbated by a detrimental effect of abdominal obesity and HF. Finally, activation of the leptin-aldosterone-neprilysin axis appears to contribute importantly to the natural evolution of HF in T2DM patients with obesity and NT-proBNP levels require more thorough evaluation to predict HFpEF [46]. Moreover, its role in predicting a potential treatment response in T2DM patients at higher risk of HFpEF remains unclear, while NT-proBNP remains an effective tool for eligibility and enrichment for CV events mainly in established HF. However, elevated NT-proBNP levels seem to yield resembling relative risk information for HFrEF and HFpEF, T2DM and abdominal obesity, and contribute a much more discriminative value in HFpEF patients with lower NT-proBNP levels than in HFrEF patients [47].

Apelin plays a central role in insulin resistance, glucose metabolism, obesity-related inflammation, water homeostasis and osmotic regulation in T2DM [47]. Perhaps, apelin/apelin receptor modulating intracellular signal transduction pathways mediates not only a positive inotropic effect on the myocardium, but also exerts fluid retention and potentiates exacerbation of HF [48]. However, serum levels of apelin have previously exhibited its predictive potent for CV outcomes mainly in HFrEF, but not in HFpEF [25]. There is limited evidence regarding the fact that left ventricular diastolic dysfunction in T2DM patients was remarkably associated with over-expressions of several adipocytokines having pro-inflammatory properties including apelin in white adipose tissue, and with elevated levels of apelin in circulating blood [49]. Indeed, in the study we found that HOMA-IR, BMI and dyslipidemia closely related to serum apelin levels and that there was a remarkable negative association between the levels of apelin and LVEF, whereas echocardiographic parameters of left ventricular function including E/e’ and LV hypertrophy correlated negatively with circulating levels of apelin. These findings particularly coincide to the results that were previously received by Li L et al. (2006) [50], but authors investigated T2DM and obese populations without HF.

We hypothesized that apelin to NT-proBNP ratio may be more predictable that these biomarkers alone, especially in T2DM with comorbidities including abdominal obesity. Indeed, multivariate regression model yielded that the apelin to NT-proBNP ratio < 0.82 × 10^−2^ had better predictive ability to biomarkers of LV diastolic dysfunction as well as apelin and NT-proBNP alone. Therefore, we suggested that this ratio may be useful for T2DM patients receiving optimal pharmacological therapy and probably continuous monitoring for this ratio during point-of-care therapy of T2DM and HFpEF could have practical utility, especially for patients with HFpEF accompanied with hyponatremia and having lower levels of NT-proBNP, but this assumption requires being confirmed in the future. Therefore, apelin demonstrated a protective capacity against acute myocardial infarction/cerebral ischemia and the effect was mediated by preconditioning ischemia, NO production and reduction of lipid peroxidation and apoptosis; however, the results obtained in animal models need to be confirmed in clinical settings [17,51]. However, apelin promoting metabolic and functional recovery of myocardium seems to be a promising molecular target for pharmacological therapy of HF in the future [52]. Probably, the apelin to NT-proBNP ratio could be a better predictor for HFpEF in patients without congestion, while this assumption requires elucidation in larger studies [53]. Finally, practical utility of the apelin to NT-proBNP ratio needs more investigation taking into consideration of a wide implementation of SGLT2 inhibitors in the therapy of both T2DM and HFpEF/HFrEF, because these agents seem to show a favorable effect of apelin and NT-proBNP [54].

### Study Limitations

The study has several limitations, such as a retrospective single center design, and small sample size. Undoubtedly, retrospective observation seems to be an obvious limitation because it might influence variety of the biomarkers, but the study was based on the data received from patients who were under investigation by the same researcher in the single center. Therefore, it is possible that higher variability of the biomarkers are a result of a small sample size. In addition, we did not measure C-terminal cleavage of (pyr)-apelin-13 and apelin-17 along with total apelin, because it was not a purpose of the study to elucidate binding of pyr-apelin 13 and apelin 17 to their targets, such as ACE2, which may have serious importance in the larger population. Yet, we did not include in the study T2DM patients with atrial fibrillation/flatter, history of CVD, TIA/stroke to minimize a statistical bias. We believe that these limitations would not influence an interpretation of the results of the study.

## 5. Conclusions

We found that the levels of apelin was significantly increased in T2DM patients with HFpEF to non-HFpEF diabetics and apelin to NT-proBNP ratio < 0.82 × 10^−2^ was independent predictor for HFpEF in T2DM patients. This finding could open new approach for CV risk stratification of T2DM patients at higher risk of HFpEF.

## Figures and Tables

**Table 1:** Basic demographic, clinical and hemodynamic characteristics of patients’ study population.

Variables	Healthy Volunteers (*n* = 25)	Entire Patient Cohort (*n* = 76)	T2DM Patients (*n* = 76)		*p*-Value
			HFpEF (*n* = 48)	Non-HFpEF (*n* = 28)	
Age, year	48 (42–55)	51 (41–62)	52(43–62)	51(41–60)	NS
Male, n (%)	17 (68.0)	49 (64.5)	31 (64.6)	18 (64.3)	NS
Dyslipidemia, n (%)	-	62 (81.6) #	38 (79.1)	24 (85.7)	NS
Hypertension, n (%)	-	66 (86.8) #	43 (89.5)	23 (82.1)	NS
Smoking, n (%)	5 (20.0)	37 (48.7) #	21 (43.8)	16 (57.1)	0.05
Abdominal obesity, n (%)	-	34 (44.7) #	22 (45.8)	12 (42.9)	NS
Microalbuminuria, n (%)	-	23 (30.2) #	14 (29.1)	9 (32.1)	NS
LV hypertrophy, n (%)	-	60 (78.9) #	41 (85.4)	19 (67.9)	0.001
BMI, kg/m^2^	21.9 ± 0.5	25.8 ± 2.1 #	25.5 ± 2.4	26.3 ± 2.6	NS
Waist circumference, sm	75.0 ± 2.6	85.6 ± 2.90 #	85.0 ± 3.20	86.5 ± 3.10	NS
WHR, units	0.78 ± 0.02	0.86 ± 0.03 #	0.85 ± 0.04	0.87 ± 0.03	NS
SBP, mm Hg	127 ± 4	132 ± 5	130 ± 4	135 ± 5	NS
DBP, mm Hg	75 ± 3	80 ± 4	78 ± 4	84 ± 3	NS
LVEDV, mL	88 ± 4	154 ± 9 #	159 ±5	147 ± 6	NS
LVESV, mL	30 ± 3	62 ± 7 #	66 ± 4	59 ± 3	0.04
LVEF, %	66 ± 2	59 ± 6 #	58 ± 3	60 ± 2	NS
LVMMI, g/m^2^	80.7 ± 0.06	142 ± 6.12 #	149 ± 4.0	137 ± 3.0	0.02
LAVI, mL/m^2^	22 ± 4	33 ± 8	36 ± 4	30 ± 5	0.03
E/e’, unit	5.40 ± 0.10	8.90 ± 0.20 #	12.8 ± 0.10	7.2 ± 0.20	0.001

Notes: data of variables are given mean ± SD and median (interquartile range), #—significant difference between healthy volunteers and entire T2DM cohort. Abbreviations: WHR, Waist-to-hip ratio; BMI, body mass index; SBP, systolic blood pressure; DBP, diastolic blood pressure; LVEDV, left ventricular end-diastolic volume; LVESV, left ventricular end-systolic volume; LVEF, left ventricular ejection fraction; LVMMI, left ventricle myocardial mass index, left atrial volume index, LAVI; left atrial volume index; E/e’, early diastolic blood filling to longitudinal strain ratio; NS, not significant.

**Table 2:** Biomarkers in individuals enrolled in the study.

Variables	Healthy Volunteers (*n* = 25)	Entire Patient Cohort (*n* = 76)	T2DM Patients (*n* = 76)		*p*-Value
			**HFpEF (*n* = 48)**	**Non-HFpEF (*n* = 28)**	
eGFR, mL/min/1.73 m^2^	108 ± 5.10	83 ± 6.0	81 ± 4.2	86 ± 3.5	NS
HOMA-IR	1.53 ± 0.30	7.65 ± 3.7 #	7.90 ± 3.0	7.15 ± 2.4	NS
Fasting glucose, mmol/L	4.22 ± 0.70	5.84 ± 1.2 #	5.70 ± 1.5	5.92 ± 1.3	NS
Creatinine, mcmol/L	52.5 ± 9.15	98.4 ± 11.60	103.7 ± 9.8	95.1 ± 10.4	NS
HbA1c, %	4.20 ± 0.95	6.65 ± 0.04 #	6.54 ± 0.03	6.70 ± 0.05	NS
TC, mmol/L	4.6 ± 0.09	6.39 ± 0.04 #	6.37 ± 0.68	6.42 ± 0.55	NS
HDL-C, mmol/L	1.2 ± 0.03	0.95 ± 0.21 #	0.97 ± 0.22	0.93 ± 0.24	NS
LDL-C, mmol/L	2.8 ± 0.05	4.43 ± 0.20 #	4.42 ± 0.12	4.51 ± 0.15	0.042
TG, mmol/L	1.3 ± 0.04	2.26 ± 0.04 #	2.23 ± 0.19	2.30 ± 1.12	NS
hs-CRP, mg/L	3.21 ± 0.25	6.92 ± 1.03 #	7.56 ± 0.94	6.25 ± 0.42	NS
hs-cTnT, ng/mL	0.02 ± 0.20	0.09 ± 0.42 #	0.12 ± 0.36	0.07 ± 0.24	0.046

Notes: data of variables are given mean ± SD and median (interquartile range), #—significant difference between healthy volunteers and entire T2DM cohort. Abbreviations: HbAc1, glycosylated hemoglobin; eGFR, estimated glomerular filtration rate, TC, total cholesterol; LDL, low density lipoproteins; HDL, high density lipoproteins; TG, triglycerides; hs-CRP, high sensitive C-reactive protein; hs-cTnT, high sensitive cardiac troponin T; NS, not significant.

**Table 3:** Predictors for depending variable (HFpEF) in T2DM populations. The results of the univariate and multivariate log regression analysis.

Variables	Depending Variable: HFpEF					
	Univariate Log Regression		Multivariate Log Regression			
	OR	95% CI	*p*-Value	OR	95% CI	*p*-Value
Unadjusted log regression						
Apelin to NT-proBNP ratio < 0.82 × 10^−2^ units	1.32	1.12–2.12	0.001	1.44	1.18–2.77	0.001
LV hypertrophy	1.16	1.10–1.19	0.046	1.03	1.00–1.05	0.14
LVEF	1.03	1.00–1.06	0.62	-		
BMI > 34 кг/м^2^	1.09	1.02–1.14	0.044	1.07	1.01–1.10	0.036
Apelin > 4.5 ng/mL	1.06	1.01–1.10	0.046	1.04	1.01–1.07	0.040
NT-proBNP > 458 pmol/mL	1.24	1.06–1.33	0.001	1.17	1.02–1.26	0.042
Age	1.03	1.02–1.05	0.048	1.03	1.00–1.04	0.16
Smoking	1.04	0.98–1.07	0.92	-		
E/e’ > 11 units	1.12	1.06–1.20	0.001	1.04	1.01–1.06	0.044
LAVI > 34 mL/m^2^	1.20	1.11–1.36	0.001	1.06	1.02–1.13	0.042
SGLT2i	0.98	0.95–1.05	0.92	-		
ACEI/ARBs	0.99	0.91–1.09	0.93	-		
Obesity-adjusted log regression						
Apelin to NT-proBNP ratio < 0.82 × 10^−2^ units	1.37	1.12–3.15	0.001	1.44	1.18–2.77	0.001
LV hypertrophy	1.04	1.03–1.07	0.046	1.02	1.00–1.04	0.72
E/e’ > 11 units	1.02	1.00–1.05	0.92	-		
LAVI > 34 mL/m^2^	1.08	1.01–1.12	0.050	1.08	1.00–1.10	0.80

Abbreviations: NT-proBNP, N-terminal prohormone of brain natriuretic peptide, LVEF, left ventricular ejection fraction; LVMMI, left ventricle myocardial mass index, left atrial volume index, LAVI; left atrial volume index; E/e’, early diastolic blood filling to longitudinal strain ratio; BMI, body mass index; ACEI, angiotensin-converting enzyme inhibitor; ARBs, angiotensin-II receptor blockers; SGLT2i, Sodium-glucose cotransporter 2 inhibitors; NS, not significant.

## Data Availability

Not applicable.
